# Development of palliative care attitude and knowledge (PCAK) questionnaire for physicians in Kuwait

**DOI:** 10.1186/s12904-019-0430-9

**Published:** 2019-06-06

**Authors:** Ameena Mohammed Al-Ansari, Saleem Nawaf Suroor, Sobhi Mostafa AboSerea, Wafaa Mostafa Abd-El-Gawad

**Affiliations:** 1 Palliative Care Center, Al-Sabah Health Area, Al-Shuwaikh, Kuwait; 2 Geriatrics and Gerontology department, Faculty of Medicine, Ain Shams University, Al- Abbasseya, Cairo, Egypt

**Keywords:** PCAK, Palliative care, Attitude, Knowledge, Kuwait, Physicians

## Abstract

**Background:**

Over the past five decades, palliative care has changed from helping patients at the end of life into a highly dedicated service focused on delivering supportive care to patients with life-limiting illnesses throughout the disease trajectory. To date there is no common agreement on universally applicable measurement tool to know the areas of weakness in physicians’ understanding of palliative care and identifying misconceptions about palliative care. This paper describes the development of a reliable and valid questionnaire to provide a measure of the attitude and knowledge of physicians toward palliative care (PCAK).

**Methods:**

Item pool was generated paying particular attention to content and face validity. The initial version of the questionnaire was piloted and assessed based on psychometric criteria. Items which did not reach acceptable validity were excluded, and the final 37 item version was administered to two groups differing in their palliative care attitude and knowledge on two occasions to assess the construct validity and test-retest reliability. Two hundred thirty two physicians working in primary care clinics and general hospitals completed the questionnaire at the piloting stage. The final version (PCAK) was administered to 35 oncologists and 76 physicians. SPSS v20 was used for statistical analysis.

**Results:**

Of the Pilot study, 20 items were excluded because didn’t meet the criteria for item difficulty and discrimination. Item-to-total-score correlations (r) was ranging from 0.347 up to 0.806. Internal consistency (Cronbach’s alpha) was high ranging from 0.636 to 0.824. While testing the final PCAK, oncologist scored consistently higher than the other physicians on all sections of the questionnaire (*P* < 0.001) suggesting good construct validity. Test to retest reliability for each section was very high, ranging from 0.879 to 0.97 and the overall reliability was 0.95. The internal consistency reliability of each section was very good ranging from 0.681 ± 0.893.

**Conclusion:**

The findings demonstrate that PCAK meets psychometric criteria for reliability and construct validity. It provides a useful scale to assess the attitude and knowledge of physicians about palliative care helping in planning of educational programs for physicians.

**Electronic supplementary material:**

The online version of this article (10.1186/s12904-019-0430-9) contains supplementary material, which is available to authorized users.

## Introduction

Palliative care is a relatively new emerging subspecialty worldwide in general and Kuwait in particular [1]. Over the past five decades, palliative care has changed from helping patients at the end of life into a highly dedicated service focused on delivering supportive care to patients with life-limiting illnesses throughout the disease trajectory [2]. Most of those people have life threatening illnesses and usually suffer from multiple symptoms such as pain and dyspnea which affect their quality of life [2, 3].

Many studies showed that integrating palliative care early in the disease trajectory can result not only in a good control of such symptoms and better quality of life for those patients and their families [3–6] but also in their illness perception [5–7], goals of care discussion, acceptance of advanced care planning, and overall survival [7–10]. Moreover it decreases emergency department visits and costs of care [2, 3].

Furthermore, a great agreement nationally and internationally is given for the delivery of such care in a high quality manner. However, it is acknowledged that the increasing number of people seeking palliative care will make it difficult for palliative care teams to engage with every hospital and primary care clinic to manage patients and their family members [8, 9]. Building bridges between palliative medicine and other medical specialties especially family and emergency medicines will help in raising the physicians’ awareness about the importance of palliative medicine and their educational needs. This will be directly translated into improving the care and the quality of life of their patients. It also will ease greatly in closing the gap of patients in Kuwait who need palliative care and yet do not get it.

Many tools had been developed to measure changes in physicians and nurses attitudes, knowledge and skills. To date there is no complete agreement on single applicable measurement tool [11–13]. Most of the tools available either focused on attitude and competence in dealing with death and dying such as Frommelt Attitudes Towards Nursing Care of the Dying (FATCOD) scale [14] and the Self-Competence in Death Work Scale [15] or to measure knowledge for nurses such as nurses’ knowledge of palliative care (PCQN) [16] and The palliative care knowledge test (PCKT) [17].

Unfortunately, no valid and reliable tool is available to measure both attitude and knowledge in palliative care together with self-efficacy or knowledge for non-palliative physicians.

So, our aim was to develop palliative care attitude and knowledge questionnaire (PCAK) for non-palliative physicians to assess their attitude and basic knowledge towards palliative care. The questionnaire focused on delivering supportive care rather than end of life care. The following criteria were considered while developing the questionnaire; using simple clear language, shortness of the questionnaire to increase the compliance and ease the administration, specific to cover the topics that crucial to physicians’ palliative care practice and finally the validity and reliability of the questionnaire.

## Methods

### Development of the preliminary questionnaire

#### Developing the questionnaire item pool

An advisory committee of palliative care co-coordinators provided direction throughout the entire development process through a series of focus groups. Committee members were palliative medicine specialist experienced in palliative care with responsibilities for education and training. Their responsibilities entailed assessing the awareness and attitude of other medical specialties, identifying aspects of knowledge that were central to palliative care practice, determining the format to be used for the tool, specifying the appropriate level of difficulty for items, and generating the items [16, 18].

On the basis of the review of the tools available in the current literature and how they created by their developers either about attitude [13–15, 19, 20] or knowledge [13, 16, 17, 21] towards palliative care, it was decided to divide the questionnaire into two main sections; the first section was to assess the attitude of physicians and the second section was to assess self-knowledge (self-efficacy) and aspects of the basic knowledge that were central for palliative care practice by any non-palliative physician such as the principles of palliative care, symptoms assessment and management, and the use of painkillers.

#### Generation and pre-testing of items

Members of the advisory committee generated a pool of approximately 102 items covering all dimensions [16, 18]. For the attitude, 33 items were identified and measured through Likert scale [22–24]. It helped to quantify subjective preferential opinion, attitude, thinking and feeling in a scientifically accepted, validated and reliable manner [25–27]. We used Likert 5 points symmetrical scale. In many studies, five points scale is comprehensible, enabling respondents to accurately express their views [24, 27]. Participants were asked to show their level of agreement (from strongly disagree to strongly agree) with the given statement (items) on a metric scale. Here all the statements in combination reveal the specific dimension of the attitude towards the palliative care, hence, necessarily inter-linked with each other [26–28].

Attitude was defined as a system of beliefs and knowledge that everyone has got or has learned throughout his lifetime [29]. Health care providers’ attitude toward caring patients with life threatening disease may have an important influence on the quality of care they provide [20]. Dimension of self-knowledge included 7 items and measured by 5 points Likert scale ranging from excellent, very good, good, weak, to none. Self-efficacy was defined by Bandura, 1994 as ‘people beliefs about their abilities to make designated levels of performance that implement influence over events that affect their lives’ [30] while self-knowledge was defined as ‘competence to perform certain procedure’ [16].

Dimension of basic knowledge that where central to palliative care practice reflects the philosophy and principles of palliative care, the management and control of pain and other symptoms, and use of painkillers.

Sixty two items were identified with five response alternatives consisting of the correct response, three distracters and an ‘I don’t know’ alterative aiming to distinguish between lack of information and misinformation as well as to reduce guessing [31]. Items were generated from the literature with expert advice from experienced palliative medicine specialists based on their knowledge and real life experiences with palliative care patients. It was believed that this process served to maximize the content validity of the questionnaire to ensure that items selected were representative of the whole area of attitude and knowledge being measured.

Knowledge was defined as ‘knowing something with familiarity that acquired through experience such as understanding of a science or technique’. [32] While Ross et al. [16] defined palliative care knowledge as ‘understanding of death and dying, symptom management, medications and any intervention needed for those patients care’. We meant by basic knowledge minimal knowledge needed in physicians to deal effectively in different clinical situation commonly seen in those patients.

We omitted any items related to death or dying as our aim to shift the focus of palliative care from only caring of patients at the end of life to the delivery of highly specialized supportive care to any patients with life threatening-illness through the disease trajectory.

To ensure high face validity and the representation of a reasonably valid sample of items from the substantive areas of interest, items were then reviewed by all members of the advisory committee and other specialists in palliative care to select the best in terms of clarity and relevance of the questions, accuracy of the palliative knowledge being tapped, doubling or closeness of the items, and interpretability [16, 18]. This process reduced the number of items to 46 as a preliminary palliative care attitude and knowledge questionnaire with five response alternatives.

A number of demographic questions were added to the questionnaire to characterize the respondents. It includes 11 items about sex, age, nationality, educational level and qualification, specialty, place of work, position “job title”, years of experience, any formal palliative care training, and previous discussion with the patients or their families. Now, the preliminary instrument was then ready for piloting in a sample of physicians and included 57 items [16, 18].

### Evaluation of the preliminary questionnaire “pilot study”

Five hundred questionnaires were distributed to the physicians working in primary care clinics and general hospitals all over Kuwait. The questionnaire was distributed manually for each clinic or hospital with the request that they complete and return them and add any comments that might occur to them. Of the 500 questionnaires, only 46.4% (*N* = 232) were returned and completed. The results of the pilot study were analyzed both quantitatively for item difficulty, item discrimination and internal consistency, and qualitatively which involves looking at comments made by respondents [16, 18]. This leaded to dropping of nearly one-third (*n* = 20) of the original items.

### Evaluation of validity and reliability of the final questionnaire (PCAK) Additional files: 1 and 2

Based on the analysis described above, the number of items was reduced to 37. The next step was to test construct validity [33, 34] of the final version by administering it to two groups known to differ in their attitude and knowledge toward palliative care. The first group was oncologists working in Kuwait Cancer Control Center and consist of 47 and other group was physicians working in primary care clinics and general hospitals and consist of 82. This ensured that one group had a better attitude and knowledge (the oncologists) than the other group (other physicians), while other demographic characteristics were fairly similar for both groups.

PCAK was administered on two separate occasions, with an interval of 2 weeks between them. 2 weeks were expected to be long enough for participants to have forgotten their original responses, but not sufficiently long for much real change in their attitude and knowledge towards palliative care. Participants were not aware of the intended second administration at the time of the first [18]. The responses from the first administration were used to assess construct validity and internal consistency. The two sets of responses were used to measure test-retest reliability [33, 34].

### Ethical consideration

The approval of the ethical committee of the Ministry of Health was taken prior to the study. Informed written consent was obtained from all participants. The aim of the study and expected outcomes were explained with guaranteeing of the privacy of the data.

### Statistical analysis

All data manipulation and analysis were performed using the SPSS (Statistical Package for Social Science) SPSS version 20. *P*-values less than 0.05 were regarded as a sign of statistical significance. Categorical variables were represented as numbers and percent while continuous variables were represented as means and standard deviations or medians and interquartile ranges as appropriate. Chi-square test or Fisher’s Exact when appropriate was used to compare between qualitative variables. Independent t-test was used to compare the quantitative variables between two groups.

#### Regarding pilot study

The results were analyzed both quantitatively (for item difficulty, item discrimination and internal consistency) and qualitatively (which involved looking at comments made by respondents) [18]. For item difficulty, According to Kline (2000) [33] items are not useful if they are answered correctly by more than 80% or fewer than 20%. Pre-tests using that item run the risk of a ceiling effect in which performance on the pretest cannot be improved upon. For item discrimination, Pearson correlation was used to compare each item in attitude or knowledge with each subtotal score. An item-to-total-score correlation < 0.2 was rejected to discriminate between people with different levels of knowledge or attitude during testing of the questionnaire in the pilot study [33, 35]. Internal consistency using Cronbach’s alpha was measured separately for the different sections, each of which was tapping a different area; attitude, self-knowledge, and general knowledge. The minimum requirement for internal consistency has been accepted as 0.6 or more for research purpose [33]. Comments made by respondents were carefully revised and some changes were done upon.

#### Regarding the final questionnaire (PCAK)

The results of final survey were tested for construct validity by comparing two groups of different knowledge [33, 34] and attitude (oncologists and other physicians). Chi-square test or Fisher’s Exact when appropriate was used to compare between qualitative variables. Independent t-test was used to compare the quantitative variables between two groups.

Test-retest reliability was done to verify that the results produced were consistent over time. Paired t-test was used to compare the response of the same group before and after 2 weeks. More than 0.8 was considered accepted cut point for reliability and consistency over time. Dates of birth were used to match the two sets of questionnaires. Internal consistency was tested using Cronbach’s alpha as above. The responses from the first administration were used to assess construct validity and internal consistency. The two sets of responses were used to measure test-retest reliability [33–36].

Factor analysis was performed and repeated many times throughout the development of the questionnaire. Kaiser-Meyer-Olkin Measure of Sampling Adequacy more value > 0.5 is acceptable and indicating that pattern of correlation between items relatively compact and suitable for factor analysis. Bartlett’s Test of Sphericity was done. Extraction method used was principal axis factoring [37]. Variables were exclude if they have low Communalities (< 0.4) as it means that the variable didn’t contribute much to measuring the underlying factors.

Scoring of attitude responses (11 items): strongly disagree (1), disagree (2), not sure (3), agree (4), strongly agree (5). So, the score ranged from 11 to 55 points so the difference (44 points) was divided into three equal parts for scoring. Negative attitude if the participant scored = < 25, uncertain attitude if scored > 25 but < 41, positive or favorable attitude if scored > =41. Regarding self-knowledge, 5 points likert scale was used. Participant for each item was scored (5) for excellent response, (4) for very good, (3) for good, (2) for weak and (1) for none. Regarding basic knowledge scoring; each correct answer was scored one and wrong answered was scored zero. Poor knowledge was calculated if participant scored less than 50% of the total score (12 points) (= < 5 points), fair knowledge if > = 50% to = < 75% (6–9 points), good knowledge if scored > 75% (> = 10 points).

Although by factor analysis, the weight of each item in subscale analysis was not equal and some items were weighting more than others but from clinical point of view, we considered each item is important and relevant and has same weight like others in each subscale.

## Results

### Pilot study

Of five hundred questionnaires distributed, only 232 (46.4%) physicians returned the completed questionnaire. Men were 59.9% (*n* = 139), their mean age 41.93(10.32), the demographic characteristics were shown in Table 1. For item difficulty, 17 items did not meet these criteria but 3 items were retained on the grounds of content validity as they were considered to be testing an essential aspect of attitude or knowledge not covered elsewhere in the questionnaire.

**Table 1:** General description of the pilot study

		Total (*N* = 232)
Age		41.92 ± 10.32
Sex	Males	139(59.9%)
	females	93(40.1%)
Nationality	Kuwaiti	46(19.8%)
	Non-Kuwaiti	186(80.2%)
Qualification	MBBS	91(39.2%)
	Master	101(43.5%)
	MD, MRCP/MRCS	40(17.3%)
Specialty	ER	49(21.1%)
	Family Medicine	45(19.4%)
	Internal Medicine	26(11.2%)
	GP	77(33.2%)
	Surgery	6(2.6%)
	Others	29(12.5%)
Years of experience		15.34 ± 9.36
Place of work	Primary care clinics	160(69%)
	Hospitals	72(31%)
Discussion about palliative care	No patients	156(67.2%)
	1 to 5 patients	54(23.3%)
	6 to 10 patients	12(5.2%)
	11 to 15 patients	3(1.3%)
	> 15 patients, families	7(3%)
Receiving any formal palliative training	Yes	12(5.2%)
	No	220(94.8%)
Attitude scores	Total score	34.75(7.4)
	Favorable attitude	52(22.4%)
	Unfavorable attitude	30(12.9%)
	Uncertain	150(64.7%)
Basic knowledge	Total score	5.64 ± 2.06
	Good knowledge	4(1.7%)
	Fair knowledge	115(49.65)
	Poor knowledge	113(48.7%)

For item discrimination, six items were excluded due to poor discrimination (r < 0.02). Other item-to-total-score correlations (r) were ranging from 0.347 up to 0.806. In attitude section, it was 0.534, Self-knowledge part, it was 0.806, and in basic knowledge, it was 0.520. Furthermore, all items had a statistically significant relationship with the subtotal score (*p* value < 0.001). Consequently, these findings provided evidence that PCAK is reflective of an individual’s attitude and level of knowledge about palliative care. Internal consistency was measured separately for the different sections using Cronbach’s alpha. It was high ranging from 0.636 to 0.824. Some changes to wording were made in response to comments written on the questionnaires, in order to reduce uncertainty and maximize the clarity of the questions. Finally, one-third (*n* = 20) of the original items was dropped because they were unclear or because they didn’t meet the criteria for item difficulty or discrimination.

### Final questionnaire (PCAK) Additional file: 3

**Fig. 1:**
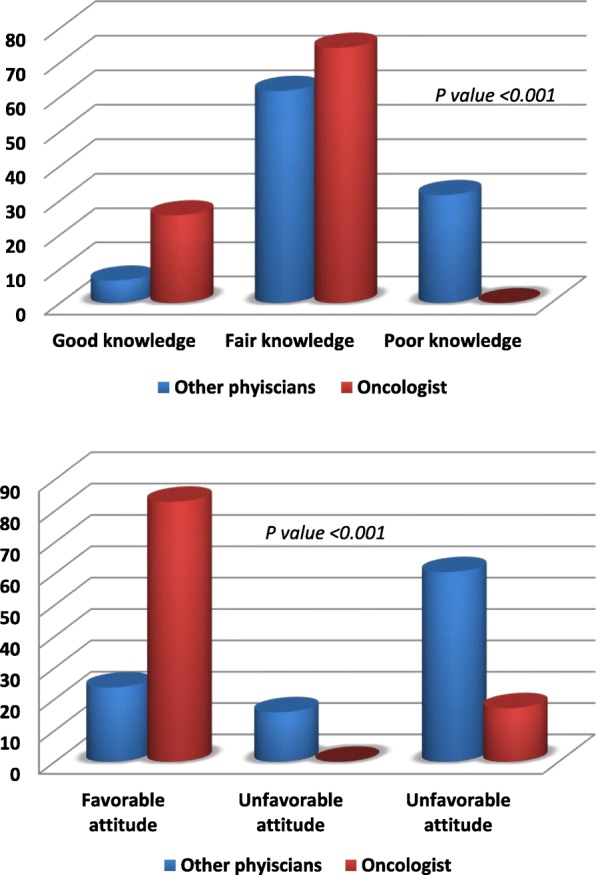
the differences in attitude and knowledge between oncologists and other physicians from other specialties

Based on the analysis described above, the number of items was reduced to 37. PCAK was administered on two separate occasions, with an interval of 2 weeks between them. The compliance was fair (47 oncologists and 82 physicians), 111 (86.1%) of those completed the questionnaire twice (35 oncologists and 76 physicians). Differences in age and sex between the two groups were not significant. The demographic characteristics of the two groups were shown in Table 2. Figure 1 showed that the oncologist scored consistently higher than other physicians in all sections of the questionnaire (*P* < 0.001). The questionnaire therefore met the criterion for construct validity. As shown in Table 3, test to test reliability for each section was very high, ranging from 0.879 to 0.97 and the overall reliability was 0.95. The internal consistency reliability of each section was established using Cronbach’s alpha (0.601 to 0.806). Correlations were very good ranged from 0.394 to 0.893. Interestingly, there is positive association between the attitude and basic knowledge either in total scores (*p* value < 0.001) or between the subgroups (p value 0.027). (Table 4).

**Table 2:** Comparison between the oncologists and other physicians in demographic characteristics

		Oncologists *N* = 35	Other Physicians *N* = 76	P value
Age		37.7(5.83)	40.95(9.94)	0.077
Sex	Male	25(71.4%)	50(68.1%)	0.664
	Female	10(28.6%)	26(34.21%)	
Nationality	Kuwaiti	2(5.7%)	8(10.5%)	0.500
	Non-Kuwaiti	33(94.3%)	68(89.5%)	
Qualification	MBBS& Master	27(77.1%)	65(86.7%)	0.269
	MD, MRCP/MRCS	8(22.95%)	10(13.3%)	
Years of experience		11(8–15)	14(7–14)	0.077
Receiving any formal palliative training	Yes	4(11.43%)	1(1.32%)	0.049
	No	31(88.66%)	75(98.68%)	

Physicians (internist, primary care physicians, emergency physicians...)

**Table 3:** Internal consistency, Test retest reliability, Item to total correlation scores of attitude and knowledge

		Total *N* = 111	Oncologists *N* = 35	Other Physicians *N* = 76
Internal consistency Cronbach’alpha	Attitude section	0.806	0.816	0.725
	Self-knowledge	0.731	0.615	0.687
	Basic knowledge	0.601	0.602	0.604
Test retest reliability	Attitude section	0.997	0.996	0.998
	Self-knowledge	0.980	0.978	0.951
	Basic knowledge	0.936	0.879	0.932
Item to total correlation (r)	Attitude section	0.394–0.749	0.415–0.893	0.445–0.61
	Self-knowledge	0.747–0.836	0.533–0.822	0.763–0.804
	Basic knowledge	0.402–0.676	0.395–0.724	0.435–0.642

**Table 4:** Relationship between the attitude and knowledge

	Knowledge			
	good	Fair	Poor	P value
Favorable attitude	10(71.4%)	32(43.8%)	5(20.8%)	0.027
Unfavorable attitude	0	7(9.6%)	5(20.8%)	
Uncertain	4(28.6%)	34(46.6%)	14(58.3%)	
Total		r = 0.321		< 0.001

**Fig. 2:**
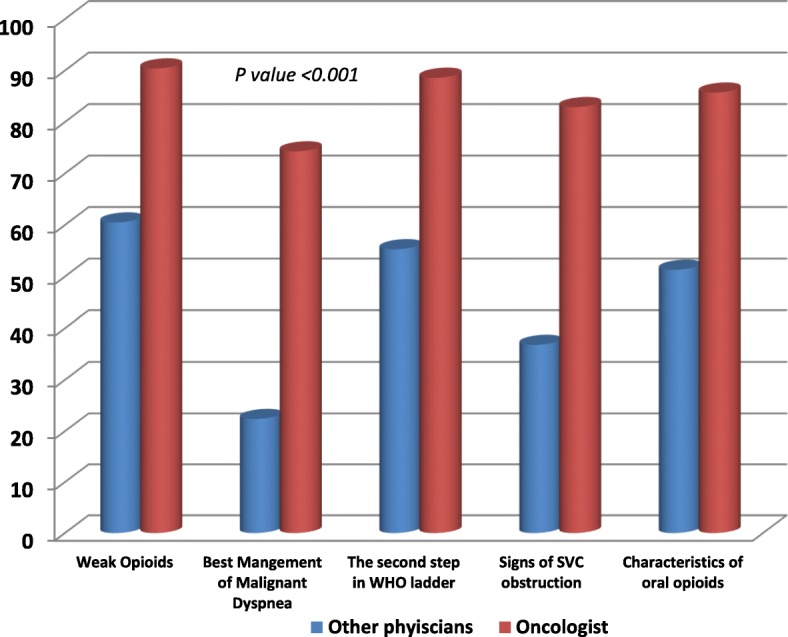
Comparison between the answers of oncologists and other physicians in a sample of questions from Knowledge Section

**Fig. 3:**
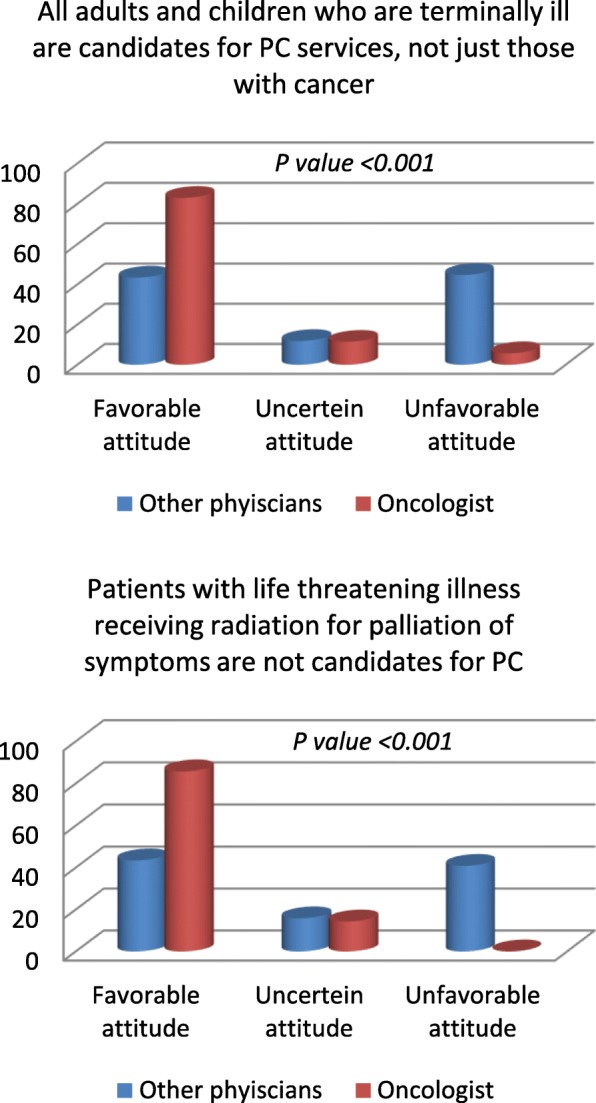
Comparison between the responses of oncologists and other physicians in a sample of from Attitude Section

Comparisons between the responses of oncologists and other physicians in a sample of items from knowledge and attitude sections were shown in Figs. 2 and 3.

Participants replied back positively during taking the questionnaire and were interested in the correct answers (91.5%, 43 out of 47 of oncologists and 89%, 73 out of 82 of physicians). Although not quantified, their verbatim responses regarding the usefulness of the questionnaire suggest that the PCAK is thought to provoke brain storming and eye-opening.

Final factor analysis for the questionnaire had acceptable results. Kaiser-Meyer-Olkin Measure of Sampling Adequacy was 0.699, Bartlett’s Test of Sphericity was significant (*p* value< 0.001), and Communalities was ranging from 0.467–0.730.

For Attitude, Self-knowledge (self-efficacy) and Knowledge subscales factor analysis, Kaiser-Meyer-Olkin Measure of Sampling Adequacy were 0.736, 0.678, 0.742, Communalities were from 0.516 to 0.827, 0.560 to 0 .636 and 0.487 to 0.701 respectively.

## Discussion

The effective measurement of knowledge and attitude of the physicians is an important component of the evaluation of education and practice. Such measurement can be used in a variety of purposes such as the assessment of learning needs and the evaluation of any educational program or service.

According to World Health Assembly (WHA), it is crucial that health educators and administrators to include content related to palliative care in most educational programs and to be systematically addressed rather than incidentally included. There is a growing evidence of the influence of palliative care services in providing compassionate and competent care for the patients [1, 38].

Unfortunately, most of the studies were done to assess the attitude and knowledge in palliative care in nurses [39–44] rather than physicians [40, 45]. Again most of them assessed the attitude toward death and dying rather than palliative care and its benefits [39, 41–43]. Many of them used self-administered questionnaires such as Palestine [44], Lebanon [40], India [43] and Thialand [45]. While other 2 studies from Saudi Arabia [42] and Ethiopia [41] used a combination of the PCQN [16] and FATCOD [14]. In Ireland, the authors used the PCQN [16] and the Thanatophobia scale (TS) [46] while in India the authors used FATCOD [14] to assess the attitude.

Interestingly, we found a positive association between attitude and basic knowledge scores. Many studies reported a significant correlation between the level of knowledge and attitudes towards palliative care. This is highlighting that as participants’ level of knowledge increased, attitudes become more positive either setting in hospitals such as in Lebanon [40], India [43], Ethiopia [41] and Saudi Arabia [42] or primary care setting such as in Thailand [45]. It is a part of human nature that the degree and complexity of knowledge affect their attitudes and in turn their behavior [47].

Unfortunately, there is no reliable or validated questionnaire to assess both attitude and knowledge of physicians towards palliative care. So, our aim was to develop a psychometrically reliable and validated tool (PCAK) to assess physician’s needs. PCAK is 37-item test and takes around 20 min to administer. It is an easy short questionnaire with clear language.

In our study, respondents varied in their responses to the PCAK according to their specialty. Oncologists had significantly better attitude and knowledge toward palliative care (because palliative care is a core part of their education and work) than other physicians from other specialties such as primary care, internal medicine or emergency medicine physicians. This confirmed the construct validity of the questionnaire.

The PCAK was also intended to identify misconceptions about palliative care such as palliative care focused on the quality of life and comfort care not only care of dying patients. We intentionally dropped any items related directly to death either in attitude or knowledge. The questionnaire emphasized on the critical opioid use for refractory dyspnea [47, 48]. The PCAK was developed to stimulate discussion about palliative care especially if used prior to any educational activity or program as it would result in more willingness to contribute in discussion and generate greater receptivity to the educational materials [49–51]. Again it can be applied post activity to document the change in their knowledge and attitude and at the same time to evaluate the efficiency of the training and educational program. Finally, PCAK can help to fill the gap in the tools available for teaching and research purposes.

### Limitations

Despite Its potential use, it is not designed to provide comprehensive assessment of higher levels of knowledge associated with expertise in palliative care practice. It focuses on the primary level of information that would normally be found in introductory courses, workshops and programs. Unfortunately, palliative care curriculum is not an integral part of the undergraduate medical students. We omitted any items related to death or dying as our aim to change the focus of palliative care from only helping patients at the end of life to delivery of supportive care to any patients with life threatening-illness through the disease trajectory. Furthermore, many tools available either to assess attitude toward death such as FATCOD scale [14] or to measure the competence in dealing with death and dying such as the Self-Competence in Death Work Scale [15].

## Conclusion

In conclusion, the palliative care attitude and knowledge questionnaire (PCAK) appears to be a promising reliable and valid tool for assessing attitude and knowledge, stimulating discussion and identifying misconceptions among physicians.

The questionnaire can be useful as a teaching tool that helped in the evaluation of educational programs related to the provision of palliative care. It aims at improving the quality of education received by health care providers and ultimately the quality of palliative care offered for the palliative care patients.

## Additional files

Additional file 1: Part 1 of Palliative Care Attitude and Knowledge Questionnaire (PCAK) (DOCX 29 kb)

Additional file 2: Part 2 of Palliative Care Attitude and Knowledge Questionnaire (PCAK) (DOCX 18 kb)

Additional file 3: Palliative Care Attitude and Knowledge Questionnaire (PCAK) row data to be opened by Statistical Package for Social Science (SPSS) program (SAV 19 kb)

## Data Availability

The datasets used and/or analyzed during the current study are available as a Additional files 1, 2 and 3.

## Abbreviations

FATCOD: Frommelt Attitudes Towards Nursing Care of the Dying scale
PCAK: Palliative care attitude and knowledge questionnaire
PCKT: The palliative care knowledge test
PCQN: Palliative care quiz for nurses
TS: Thanatophobia scale
WHA: World Health Assembly
