# Supplementary material for: Development of palliative care attitude and knowledge (PCAK) questionnaire for physicians in Kuwait
Source: BMC Palliat Care. 2019 Jun 6;18:49. doi: 10.1186/s12904-019-0430-9 (PMC6555752; doi:10.1186/s12904-019-0430-9)
Supplement: Supplementary file 1 — Part 1 of Palliative Care Attitude and Knowledge Questionnaire (PCAK) (DOCX 29 kb) [file 12904_2019_430_MOESM1_ESM.docx]

**Attitude And Knowledge Of The Physicians Toward Palliative Care Questionnaire (PCAK)**

This is a survey, not a test. Your answers will help to improve palliative care services in Kuwait.

1. It is important that you complete it by yourself.

2. Your answers will remain strictly anonymous.

3. If you do not know the answer, mark `not sure' or ‘I don’t know’ rather than guess

**Participant number:**

**Section 1: Demographic Data**

**1- Date of birth: …………………..**

**2- Sex**

1. Male
2. Female

**3- Nationality: …………………..**

**4- Degree**

1. MBBScH
2. Master
3. MD
4. MRCP/MRCS

**5- Position:**

1. Trainee
2. Assistant registrar
3. Registrar
4. Senior Registrar
5. Specialist
6. Consultant

**6- Specialty:**

1. General practitioner
2. Family Medicine
3. Internal medicine
4. Emergency physician
5. Surgeon
6. Others ……………..mention……

**7- Years in practice: ………………….**

**8- Place of work:……………………**

**9- Discussions of palliative care (past three months)**

1. No patients, families
2. 1 to 5 patients, families
3. 6 to 10 patients, families
4. 11 to 15 patients, families
5. More than 15 patients, families

**10- Did you receive any formal training (workshops, lectures, teaching) in palliative care**

1. Yes 2. No

**11- If the answer of Q10 was yes, please mention for how long?......................**

**Section 3: Knowledge about Palliative Care**

**Part 1:**

1. How would you rate your experience in managing pain in cancer and palliative patients?
2. Non
3. Weak
4. Good
5. Very Good
6. Excellent
7. How would you rate your experience in managing other palliative care symptoms (constipation, nausea and vomiting, anorexia, etc.)?
8. Non
9. Weak
10. Good
11. Very Good
12. Excellent
13. How would you rate your experience in conducting family counseling and breaking bad news?
14. Non
15. Weak
16. Good
17. Very Good
18. Excellent

**Part 2:**

1. Palliative care is different from traditional care because palliative care:
2. I don't Know
3. Is curative.
4. Is equivalent to hospice care.
5. Is focused on comfort, rather than cure.
6. Is equivalent to end of life care
7. Withdraws care.
8. Which of the following members of the healthcare team are important to the delivery of palliative care?
9. I don't Know
10. Physicians
11. Nurses
12. Dietitians
13. Physical and occupational therapists
14. All of the above
15. Which of the following drugs is considered weak opioids;
16. I don't Know
17. Morphine
18. Codeine
19. Hydromorphone
20. Fentanyl
21. Oxynorm
22. Which is the most appropriate drug can be used in management of delirium in palliative care:
23. I don't Know
24. Fentanyl
25. Haloperidol
26. Midazolam
27. Zofran
28. Paroxetine
29. Dyspnea related to advanced lung cancer can be best treated by:
30. I don't Know
31. Morphine
32. Midazolam
33. Dexamethazone
34. Oxygen mask and Ventolin
35. 1 & 2
36. The second step in WHO ladder for treatment of chronic pain is:
37. I don't Know
38. Strong opioid ± adjuvant therapy
39. Non-opioid ± adjuvant therapy
40. Weak opioid ± adjuvant therapy
41. Adjuvant therapy
42. Strong opioid ± pain intervention modalities
43. Regarding Hypercalcemia in cancer patients, it:
44. I don't Know
45. Is the most common life threating metastatic disorder in cancer patients
46. Needs to be corrected if adjusted serum calcium ≥3.5 mmol/l or the patient is symptomatic.
47. Related to bone cancer only.
48. Can cause severe diarrhea.
49. All of the above
50. The hallmarks of opioid toxicity are all the following except:
51. I don't Know
52. Oxygen saturation ≤ 90%
53. Respiratory rate ≤ 10 /min
54. Pinpoint pupils
55. Sedation
56. Jerky movement
57. Signs of superior vena caval obstruction are all the following *EXCEPT*:
58. I don't Know
59. Cyanosis
60. Pulsatile distended neck veins
61. Edema in the hands
62. Dilated veins over the chest wall
63. Periorbital edema
64. Management of Catastrophic bleeding in palliative care includes
65. I don't Know
66. Midazolam
67. Vitamin K
68. Dark towel
69. Tranexamic injection in large doses.
70. 1 & 3
71. “Golden standard” treatment of metastatic spinal cord compression include the following:
72. High dose dexamethasone
73. Neurosurgical intervention
74. Chemotherapy
75. Radiotherapy
76. All of the above
77. All of the following are characteristics of oral opioid analgesics except:
78. I don't Know
79. Effective for localized and generalized pain
80. Easily administered
81. Stigma and fears associated with use
82. Ceiling effect to analgesia
83. None of the above

**Thank you very much for your time.**

**If there are any comments you would like to make about this questionnaire, please do so below, they would be very welcome.**
