# Supplementary material for: Development of palliative care attitude and knowledge (PCAK) questionnaire for physicians in Kuwait
Source: BMC Palliat Care. 2019 Jun 6;18:49. doi: 10.1186/s12904-019-0430-9 (PMC6555752; doi:10.1186/s12904-019-0430-9)
Supplement: Supplementary file 2 — Part 2 of Palliative Care Attitude and Knowledge Questionnaire (PCAK) (DOCX 18 kb) [file 12904_2019_430_MOESM2_ESM.docx]

**Section 2: Attitude toward Palliative Care**

Please tick one box per item that it mostly fit your opinion

|  | Strongly Disagree | Disagree | Not Sure | Agree | Strongly Agree |
| --- | --- | --- | --- | --- | --- |
| 1. I have been dissatisfied with the available palliative care services. |  |  |  |  |  |
| 1. I feel there is a lack of timely communication between palliative care providers and myself. |  |  |  |  |  |
| 1. I am not familiar with palliative care services in this community. |  |  |  |  |  |
| 1. I am uncertain of the length and types of coverage under the palliative care benefit. |  |  |  |  |  |
| 1. Palliative care is the right for the patient since day one diagnosis of life threatening illness as long as he/she has complex symptoms such as pain, dysnea…etc. |  |  |  |  |  |
| 1. Patients or families are unwilling or unready to elect palliative care services. |  |  |  |  |  |
| 1. All adults and children who are terminally ill are candidates for palliative care services, not just those with cancer. |  |  |  |  |  |
| 1. Patients receiving radiation for palliation of symptoms are not candidates for palliative care. |  |  |  |  |  |
| 1. Palliative care benefits include enhanced quality of life for the patient and family. |  |  |  |  |  |
| 1. Palliative care benefits include skilled care for terminally ill patients. |  |  |  |  |  |
| 1. Palliative care benefits include expert pain and symptom management. |  |  |  |  |  |
